# Development of an Efficient, Effective, and Economical Technology for Proteome Analysis

**DOI:** 10.21203/rs.3.rs-3165690/v1

**Published:** 2023-07-17

**Authors:** Yanbao Yu, Katherine Martin, Ha Le, Canyuan Yang, Guotao Lu, Xiaohui Zhang, Catherine Grimes, Zhihao Zhuang, Papa Nii Asare-Okai

**Affiliations:** University of Delaware; University of Delaware; University of Delaware; University of Delaware; CDS Analytical LLC; CDS Analytical LLC; University of Delaware; University of Delaware; University of Delaware

## Abstract

Proteomics experiments have typically high economic and technical barriers to broad biomedical scientists, which not only result in costly supplies and accessories for sample preparation but also the reluctance to adapt new techniques. In the present study, we present an effective and efficient, yet economical technology, which we call E3technology, for proteomics sample preparation. By immobilizing silica microparticles into a polytetrafluoroethylene (PTFE) matrix, we developed a novel medium, which could be used as a robust and reliable proteomics platform to generate LCMS-friendly samples in a rapid and low-cost fashion. Using different formats of E3technology, including E3tip, E3filter, E3cartridge, and E3plate, we explored a variety of sample types in varied complexity, quantity, volume, and size, including bacterial, fungi, mammalian cells, mouse tissue, and human body fluids. We benchmark their performance against several established approaches. Our data suggest that E3technology outperforms many of the currently available techniques in terms of proteome identification and quantitation. It is widely applicable, highly reproducible, readily scalable and automatable, and is user-friendly and stress-free to non-expert proteomics laboratories. It does not require specialized expertise and equipment, and significantly lowers the technical and economical barrier to proteomics experiments. An enhanced version, E4technology, also opens new avenues to sample preparation for low input and/or low-cell proteomics analysis. The presented technologies by our study represent a breakthrough innovation in biomedical science, and we anticipate widespread adoption by the proteomics community.

## Introduction

The ultimate goal of a proteomics analysis is to analyze all the proteins, the so-called proteome, of a biological sample, so the biology and/or pathology-relevant molecules and marker proteins, especially those in low abundance, could be revealed. Therefore, a major component of a bottom-up/shotgun proteomic experiment is the protein preparation, which includes cell lysis and protein extraction, protein cleanup and digestion, and peptide desalting and/or fractionation.^1^ Efficient cell lysis is critical to achieve unbiased protein extraction with a high yield. A variety of cell disruption methods, including chemical (e.g., urea, SDS, TFA, etc.) and physical based (e.g., sonication, bead-beating, homogenization, etc.), have been extensively employed by the community.^2,3^ For protein digestion, it could occur either in solution, given the proteins are fully denatured and no enzyme-interfering chemicals are present, or on solid support, such as polyacrylamide gel. Digestion of proteins in gel matrix has been one of the most traditional ways of preparing samples for MS analysis.^4^ Recently, gel-free approaches, such as filter-based reactor-type sample processing, have emerged as rapid and robust alternatives to proteomic sample preparation. Membranes,^5–8^ magnetic beads,^9^ or a hybrid format (e.g., immobilized chromatographic particles in PTFE membrane)^10^, are typically used by these filter-based methods. Their commercialization and the resulting ready-to-go products, such as Microcon and Vivacon filters, S-Trap devices, EvoSep tips and iST kits, have provided great convenience to the community and enabled wide adoption of the methods by the community.^2,11^ However, most of these commercial products are costly. For instance, compared to the DNA extraction columns that are commonly used for plasmid DNA preparation in genomic science, and are commercially available from over 20 vendors (e.g., Miniprep spin columns), the devices for proteomic sample preparation are not only limited in the market but also are three to twenty times more expensive. The high cost is likely due to the restriction of making materials, limitation of manufacturing capacity, or simply limited number of vendors competing in the market. There is an increasing need in developing and commercializing new methods that can have combined merits of cost-effectiveness, efficiency, good tolerance to detergent, and robustness.

Organic solvents have been well known to induce protein precipitation, which can be used to eliminate contaminations and purify proteins.^12^ For proteomics analysis, the precipitated proteins can be re-solubilized and subjected to in-solution digestion.^11^ Alternatively, the proteins can be precipitated directly onto magnetic beads with subsequent on-bead digestion. Such a method has been established as the single-pot, solid-phase-enhanced sample preparation (SP3) technology.^9^ However, SP3 suffers from typical concerns related to nearly all free-bead based processing methods, such as potential sample loss to tube walls and pipette tips,^14^ unintentional disruption of protein aggregates,^15^ inconsistent aliquoting of bead suspensions or insufficient distribution of beads due to rapid sedimentation,^16^ and possible cross contaminations during automation.^17^ In addition, SP3 processing requires pH adjustment, is protein concentration-dependent, and has to meet a certain bead-protein ratio, all of which increase its technical barrier for quick adaption and standardization. Interestingly, it was revealed recently that the on-bead protein aggregation is independent of bead surface chemistry.^15^ Another study by Johnston et al. further demonstrated that the solid phase could be omitted entirely, favoring a conventional “precipitation-resolubilization-in solution digestion” approach.^14^ Although the authors also investigated protein aggregation onto inert glass beads, they claimed only marginal advantages over a centrifugation-based, bead-free method.

In the present study, we set out to explore a hybrid scheme of the existing “free-bead” and “bead-free” approaches - immobilized beads. Immobilized chromatographic beads in PTFE meshwork have been well known as Empore membranes (previously owned by 3M, now by CDS Analytical). In particular, the C18-based membrane has been widely employed by the proteomics community in a format of stop-and-go-extraction tip (StageTip) for peptide desalting.^18^ Lately, membranes immobilized with other types of chromatographic beads such as strong anion exchange (SAX), strong cation exchange (SCX), and poly(styrene-divinylbenzene) reversed-phase sulfonate (SDB-RPS) were also explored for protein cleanup, digestion and fractionation.^10,19–23^ Empore membranes hold great advantages over free beads. This is because the free particles are entrapped into the PTFE matrix, so the resulting resin-containing membrane is much easier to manipulate (e.g., aliquoting, transferring, etc.). In addition, since the Empore membrane can be conveniently packed into individual filter devices or multi-well plates, the membrane-based sample preparation is readily scalable and automatable with no need of extensive method adjustment or reoptimization.^17^ Here, for the first time, we merge the Empore technology and glass beads to build a immobilized glass bead membrane, and develop an efficient, effective, and economical approach, named E3technology, for proteomic sample preparation. We benchmark its performance using different formats and a variety of sample types in varied complexity, volume and quantity. We compare them side-by-side with several established methods, and evaluate the quantitative and qualitative performance of the E3technology. We further developed an enhanced ‘single-vessel’ approach, named E4technology, to process low-input samples.

## Results

### Initial evaluation of E3technology for global proteomics analysis.

#### Design.

We first set out to examine the feasibility of using a glass bead (GB) membrane for proteomic sample preparation. We compared its performance with FASP,^6^ a commonly used filter-based method, which utilizes molecular weight cut-off membrane to retain proteins. We also carried out side-by-side comparison with another digestion method that utilizes loose GBs, the so-called SP4-GB method.^14^ In this method, the proteins are precipitated onto GBs by acetonitrile followed by on-bead digestion. In addition, we tested two different sizes of the GBs, one was in 9–13 mm range purchased from Sigma, and the other was around 30 mm from an undisclosed vendor. Initial inspection of the GB membranes revealed that the beads were held in place and embedded in the PTFE meshwork (**Supplemental Figure S1A**). A pilot digestion experiment indicated that the two GB membranes provided equivalent identification performance (**Supplemental Figure S1B**). In addition, we tested different contents of PTFE as it determines the softness of the GB membrane, which affects the integrity and robustness of the filters packed with the membrane. We noticed that, for instance, the membrane with 5% or lower PTFE content could cause sample loss or visible leakage. In this study, we used 8% PTFE and 30 mm beads to build GB membranes. Further optimization or variations could be made, if desired, to fit for different applications and purposes.

#### Qualitative assessment.

**Figure 1:**
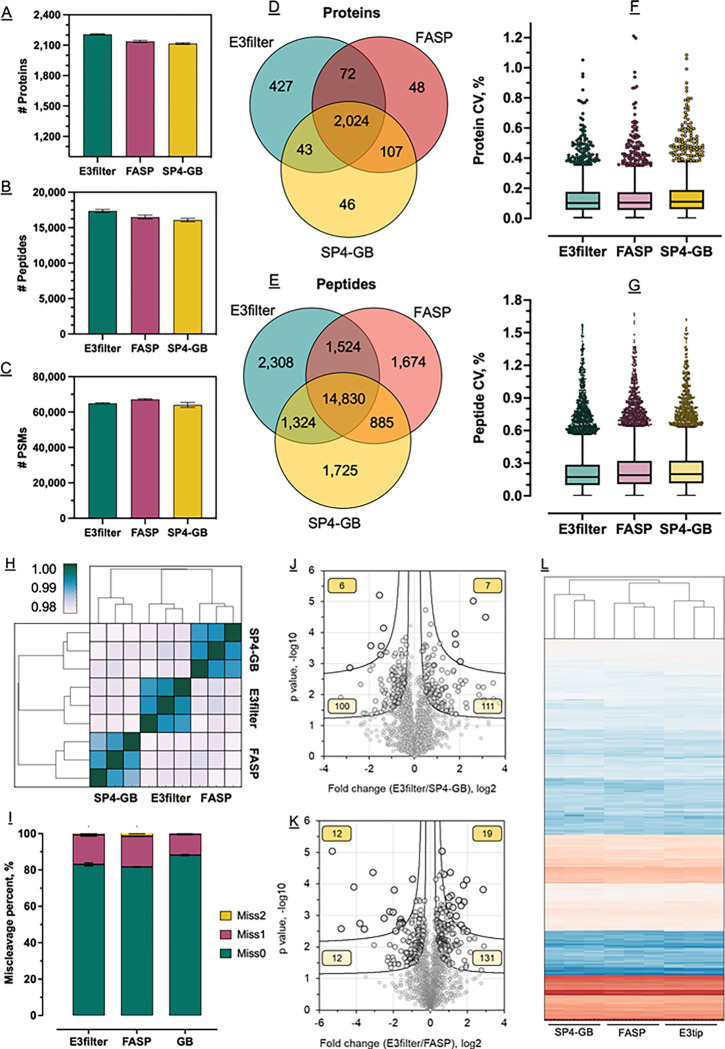
Qualitative and quantitative assessment of E3technology (E3filter) for *E. coli* proteome analysis. (A-C) Comparison of the number of proteins, peptides, and PSMs between the E3filter, FASP, and SP4-GB approaches. Error bars represent three replicates. (D-E) Overlapping analyses of proteins and peptides derived from the three methods. (F) Coefficient of variation of quantified proteins by the three methods. (G) Coefficient of variation of quantified peptides. (H) Heat map of Pearson correlation between replicate experiments and different methods. (I) Percentages of missed cleavages. (J and K) Volcano plot showing significantly differential proteins between E3filter vs. FASP and E3filter vs. SP4-GB. The two curves show FDR 0.05 and 0.01, respectively. Boxed numbers are significant proteins for each category. (L) Overall heatmap of all the replicates of the three methods.

We initially explored *E. coli* proteome to assess the qualitative and quantitative proteomic performance of the GB membrane. Here, we built a prototype E3filter by assembling the membrane into a 0.5-ml filter device as we used previously (**Online Methods**).^24^ From three independent digestion experiments, and with an input of about 20 mg *E. coli* lysate and a single-shot, 165-min LC/MS run, the E3filter was able to identify on average 2,207 proteins and 17,358 peptides, which were consistently higher than the FASP and SP4-GB approaches also tested here (Figure 1, A and B). FASP appeared to provide slightly more peptide spectrum matches (PSMs), yet they did not translate to more unique peptides. Over 93% of the protein hits and 80% of the peptide hits derived from either FASP or SP4-GB were also identified by the E3filter (Figure 1, D and E), suggesting that the on-GB membrane digestion based E3technology is not biased toward any particular proteins or protein groups. Meanwhile, the qualitative reproducibility of the E3filter was high as well. Identification overlaps between the triplicate experiments were >95% for proteins, and >85% for peptides, consistent with the performances of FASP and SP4-GB (**Supplementary Figure S2A**). We next examined the missed cleavages to assess the efficiency of on-membrane digestion. Around 83% of the peptides derived from the E3filter approach were completely digested. Although this is slightly lower than the SP4-GB method (88%), it is still better than FASP (81.5%) (Figure 1, I). This data implies that the immobilization of glass beads may have neglectable impact on the efficiency of proteolytic digestion.

#### Quantitative characterization.

We observed excellent quantitative reproducibility for the E3technology (Figure 1H). The Pearson correlation among the triplicate experiments was on average 0.9910 (±0.0011, n=3), similar to FASP (0.9907±0.0008) and slightly better than SP4-GB (0.9886±0.0019). E3technology also quantified more proteins than the other two methods. Over 95% (95.1%) of the total proteins were quantified by at least two replicates, and nearly 90% (89.5%) were quantified by all three of them, which resulted in the largest number of proteins without missing values among the three methods tested here (**Supplementary Figure 2B**). The median coefficient of variation (CV) for the E3filter was 10.2%, smaller than FASP (10.5%) and SP4-GB (11.7%), suggesting a generally small quantitative variation by E3technology (Figure 1F). Quantitation on peptide level by the E3filter showed the lowest variations as well (17.3% vs. 19% by FASP and 19.9% by SP4-GB) (Figure 1G). Overall, the profile of quantified *E. coli* proteome by the E3filter was clustered closer with FASP than SP4-GB (Figure 1, H and L), suggesting large similarities of two membrane-based methods. Although pair-wise t-test revealed some differential proteins between the methods (Figure 1, J and K), Gene Ontology analyses did not suggest any significant terms in the context of biological process and cellular compartment (data not shown).

### E3technology is multifaceted and widely applicable.

We next asked if E3technology could be applied to more complex samples other than *E. coli* proteome. Here we examined HEK293 mammalian cells, mouse kidney tissue, yeast, and human saliva with varied sample quantities and volumes. In addition to E3filters, we also tested other formats of E3technology by packing the GB membrane into pipette tips (200-ml volume, E3tip), cartridges (1–3 ml volume, E3cartridge), and a 96-well plate (500-ml volume, E3plate).

#### Mammalian cells.

**Figure 2:**
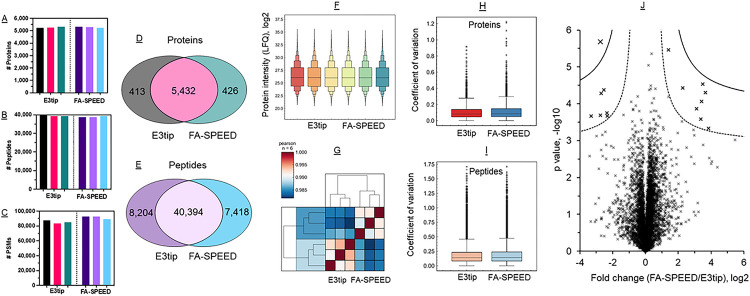
Qualitative and quantitative comparison of E3technology (E3tip) and FA-SPEED for HEK293 cell proteome analysis. (A-C) Triplicate experiments of identifications of proteins, peptides, and PSMs. (D-E) Venn diagrams of protein and peptide identifications of the two methods. (F) Overall protein intensity distribution of the two methods. (G) Heatmap of Pearson correlations. (H-I) Protein and peptide coefficient of variation. (J) Volcano plot shows quantitative comparison of the two methods.

In the context of HEK293 cells, we examined the proteomic performance of E3tips, and benchmarked against another filter-aided method (FA-SPEED) that was reported recently.^25^ In this approach, the cells were lysed with pure trifluoroacetic acid (TFA), neutralized, then precipitated with acetone followed by on-membrane (PTFE) cleanup and digestion. We applied the same protocol to the GB membrane. The experimental data indicates that from a 20-μg protein lysate of input, E3tips could identify over 5,200 and 39,000 non-redundant protein groups and peptides, respectively, as well as nearly 86,000 peptide features (peptide spectrum matches, PSMs) (Figure 2, A–C). These numbers were very reproducible among replicates, and the quantitative reproducibility was excellent as well (average Pearson correlation 0.99) (Figure 2, F and G). In terms of digestion efficiency, the percentage of missed cleavages for E3tips was about 10–13%. When compared to FA-SPEED, the identification rates were nearly the same with significant overlaps for proteins (93%) and peptides (85%) (Figure 2, D and E). Meanwhile, the quantitative correlations were consistently high for replicate experiments between the two methods. The average Pearson correlation was 0.97 for proteins and 0.93 for peptides (**Supplementary Figure S3**). These data suggest large similarities between the two membrane types. In terms quantitative variations, the median value of CV on protein level was 8.1% for E3tips and 8.5% for FA-SPEED; on peptide level was 14.0% and 14.3% for the two methods, respectively, suggesting no substantial difference (Figure 2, H and I). The minimal number of statically different proteins, 15 out of over 5,000 hits, as shown in Figure 2J, did not suggest meaningful biological significance after Gene Ontology analysis. Notably, although the FA-SPEED approach generated good quality data in our study, low recovery and reproducibility have been previously reported.^14^

#### Kidney tissues.

**Figure 3:**
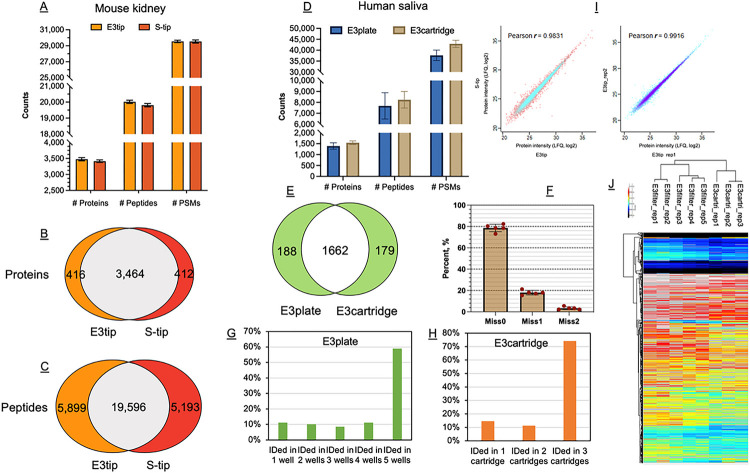
Application of E3technology to kidney and saliva specimens. (A) Histogram showing comparison of the total number of identified proteins, peptides and PSMs. Data were from triplicate experiments of each method. (B-C) Overlaps of protein and peptide identifications between the two methods (E3tip and S-tip). (D-J) Applying E3technology to saliva proteome analysis. (D), Illustration of the number of identifications by E3plate and E3cartridge. (E) Protein overlap analysis. (F) Mis-cleavage rates of saliva specimens by E3plate approach. (G-H) Frequency of protein identifications. (I) Pearson correlation of E3tip and S-tip for kidney proteome analysis. (J) Heatmap of E3plate and E3cartridge quantitation of saliva proteome.

We next evaluated the performance of E3technology using tissue samples, which tend to have wide dynamic range and high complexity.^26,27^ Here, we chose mouse kidney tissues that were adapted from our previous study,^28^ and compared E3tips with the STrap tip (S-tip) method.^8^ The latter is based on organic solvent (90% methanol) precipitation of proteins lysed in SDS buffer followed by cleanup and digestion on glass fiber filters. We applied the same processing approach to E3tips and S-tips (**Online Methods**). Our data indicate that E3tip exceeds S-tip in terms of protein and peptide identification with triplicate experiments (Figure 3A). In particular, nearly 90 and 80% of the hits were mutually identified by the two methods (Figure 3B). Quantitative correlations between the methods were high as well (Figure 3I), suggesting great similarity between the two types of membranes for proteomics analysis.

#### Human saliva.

Large-scale studies, in particular clinical proteomics and biomarker discovery science require high throughput sample processing. Thus, we investigated the potential of multiplexing the E3technology. Here, the GB membrane was casted into a deep-well 96-well filter plate. As a proof of principle, we processed human saliva specimens to demonstrate its potential for clinical proteomics analysis (Figure 3, D-H). On average, 1,400 proteins could be identified from each of the five wells, almost double the number of proteins reported by our previous study.^29^ Nearly 90% of the salivary proteins were detected in all five wells (Figure 3G). Meanwhile, the well-to-well correlation was high (Pearson > 0.94), suggesting minimal variations between the wells. The successful implementation of the E3plate provided clear evidence for its automation, which is not achievable by centrifugation-based methods, such as SP4. We next processed larger volumes (1 ml) of saliva specimens using a cartridge format of E3technology and demonstrate equivalent performance for saliva proteome identification and quantitation (Figure 3, E, H and J). This application highlighted the potential of E3technology to process diluted samples (e.g., secretome analysis) or samples with large volumes (e.g., urine).

### Enhanced E3technology enables low-input and low-cell proteomics.

**Figure 4:**
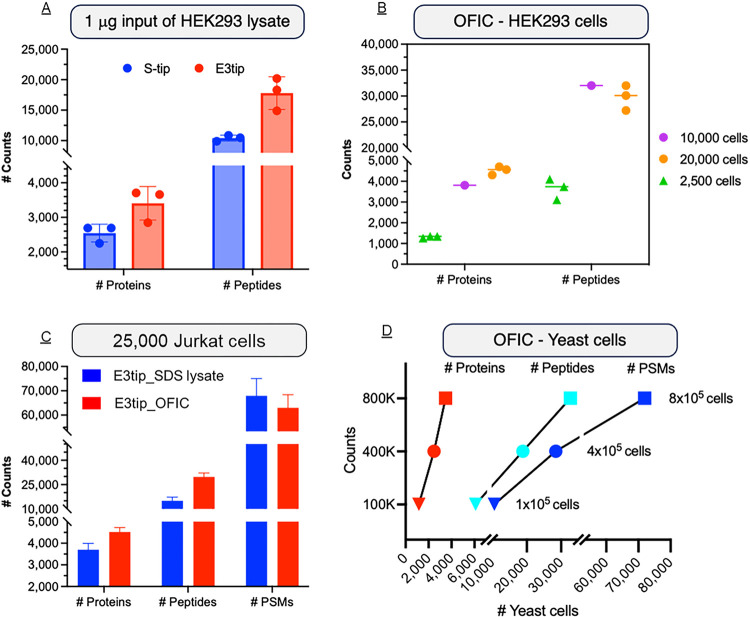
Application of E3technology to low-input samples (A) Comparison of protein and peptide identifications from 1 mg of HEK293 cell lysate. (B) Assessment of on-filter in-cell (OFIC) digestion using mammalian cells and (D) yeast cells. (C) Comparison of on-filter digestion using SDS lysate or intact cells.

Despite recent progresses of “single vessel” approaches that are supposed to reduce sample loss and increase proteomic sensitivity,^30^ analyzing quantity-limited samples such as biopsy, rare cells or precious biospecimens is still challenging. One of the possible reasons is that these methods either suffer from poor recovery when processing samples in the low μg range likely due to large processing volumes, has limitations in terms of lysing reagent selection, or require additional sample processing and transferring outside of the vessel. Here, we examined if E3technology could be used to process sub-microgram of protein samples. We took E3tip as a representative due to its relatively small contact surface and process volume. Our data showed that, from 1 mg input of whole HEK293 cell lysate, E3tip out-performed S-tip (Figure 4A), enabling the identification of over 3,700 unique proteins and 20,000 peptides, similar to the identification output of SP3 and iST methods using the same amount of lysate input.^30^

We then took a step further and asked if E3technology could be used as a “single vessel” approach to perform complete sample preparation in one device. Here, we adapted the “in-cell” digestion ideas reported recently,^31,32^ and also took advantage of the Empore technique to create a multi-functional integrated membrane. In line with the E3technology developed in this study, we mixed GBs with C18 resins and created a novel GB|C18 membrane. We then investigated if the C18-enhanced E3technology (named E4technology) could facilitate low-cell proteomics analysis. In our experiments (**Online Methods**), the cells were fixed by methanol onto the E4filters, which were then subjected to on-filter in-cell digestion (OFIC) directly. Afterward, the resulting peptides were desalted conveniently through the E4filters with C18 functionality, leading to LCMS-ready samples after lyophilization. Since no cell lysis is involved, which is known to lead to instant viscosity and sample losses to plastic surfaces (e.g., pipette tips and microtubes, and also all the processing steps were carried out in a single vessel, such an OFIC approach should minimize sample loss to the largest extend. In our initial experiment that started with roughly 25,000 Jurkat cells, we were able to identify on average over 4,300 proteins and nearly 30,000 peptides. By contrast, when the same number of cells were first lysed with SDS buffer, and then digested using the E3procedures established above, 20% and 50% less of protein and peptide hits were identified, respectively (Figure 4C). This data indicated conclusively that the conventional “cell lysis - protein digestion” methods are associated with significant sample losses. PSM.

In further experiments, our data showed that the identification rate remained consistent when the starting number of Jurkat cells reduced half (to 10,000 cells), whereas dropped to 1300 proteins and 3500 peptides when the starting material was 2,500 cells (Figure 4B). The data makes sense to this study as the LCMS setup in our experiments was not designed for single-cell proteomics. For instance, the FAIMS interface may result in some sample losses during the ion injection and gas phase fractionation process. In the meantime, we examined yeast cells following the OFIC digestion protocol, as they usually require harsh lysis conditions for protein extraction. In our experiments, interestingly, the yeast cells became completely permeable and digestible by trypsin after a simple fixing step. From around 1.6 million yeast cells (the amount of protein contents equivalent to 25K HeLa cells), we were able to identify nearly 3,500 proteins and over 32,000 peptides. When the cell number reduced by half, the identification rate of proteins and peptides were 2,472 and 18,847 (90-min run), respectively. When the cells reduced down to 160K (equivalent to 2,500 HeLa cells), the number of proteins and peptides were 1,160 and 6,097, respectively (Figure 4D).

## Discussion

Current proteomics analysis has been largely hindered by the lack of a universal sample preparation methodology that combines robustness, cost-effectiveness, and high efficiency for cell lysis, protein cleanup, and digestion. In this study, we built a novel GB membrane that is mechanically stable yet easily manageable for further assembling and building of various filter devices. We systematically investigated the feasibility of using a GB membrane for proteomic sample preparation by comparing it with several existing methods, including FASP, SP4-GB, FA-SPEED and S-Tip, while exploring a variety of sample types.

The data from our study demonstrate that the GB membrane can serve as an efficient, effective, and economical platform for sample processing prior to MS analysis. Particularly, E3filters out-performed FASP and SP4-GB methods in the context of proteome-wide identification and quantitation of *E.coli* cells. FASP has been one of the most widely adopted preparation methods in the past decade by the proteomics community,^6^ whereas the SP4-GB was reported only recently.^14^ Its principle is similar to the SP3 approach,^9^ but instead of using magnetic beads, it uses inert GBs as matrix/support for protein precipitation. Interestingly, the report claimed only marginal advantages of the GBs over a bead-free method, and stated that GBs could even be omitted altogether. We argue that the approach of using GBs for proteomic sample preparation was underestimated, and its potential could be further explored when the GBs are immobilized. The advantages of using fixed beads are obvious, as it alleviates the requirement for a minimum protein concentration and careful adjustment of protein-bead ratios and pH.^16^ It also eliminates the risks of sample loss from disaggregation and resuspension of protein aggregates and clumping beads due to fragile or dense aggregates or, non-elegant pipetting;^14^ concerns of beads that stick to pipette tips or tube walls after repeated agitation during automation are also minimized.^17^ Notably, in our SP4 experiments, we consistently detected a significant number of unprecipitated proteins from the supernatant and/or the wash solutions (data not shown). In addition, we noticed that in order to obtain a fine and uniform protein aggregates, the GBs and protein lysate have to be simultaneously added to acetonitrile solution, which otherwise will leave large buoyant (or unretained) beads on top of the solution, raising concerns of contamination or sample loss.

In FA-SPEED method, cells are lysed with pure TFA, introducing no detergents to cell lysate.^25^ The method is rapid and powerful to process many bacterial and mammalian cells, tissue samples, and microbiome standards, however, it is not necessarily universal as the authors suggested. In our experiments, TFA did not provide good protein yields when processing real fecal specimens and some filamentous bacteria such as *Leptothrix cholodnii*, whereas SDS-based buffer did (data not shown). The TFA-derived lysate is incompatible with in vitro affinity purification and protein-protein interaction studies either. In addition, the protocol involves a series of neutralization and dilution steps, thus the large final volume raises concerns of sample loss from low input or small number of cells. Notably, in this study we compared TFA lysis experiment with and without neutralization. Our data showed no qualitative and quantitative differentiation between the two procedures (**Supplementary Figure S4**), suggesting that adjustments or further optimization may be necessary in order to better fit different applications. In terms of glass fiber-based S-tip and its commercialized form, S-Trap filter, a distinct advantage of the methods is the fast liquid transfer, which makes it a rapid approach for general proteomics applications.^33^ However, unlike Empore membranes, the rigidity of glass fiber membrane is not optimal for cutting small discs and packing into pipette tips. Meanwhile, the cost of commercial S-Trap devices is comparably high. Therefore, the E3technology presents an equivalent yet economical alternative to the STrap approach.

Overall, the data from this study demonstrate that the E3technology requires only minutes of hand-on time, and is robust and compatible with a variety of upstream cell lysis conditions. Since it has no restrictions to reagents selection, and has versatile formats (e.g., E3tip, E3filter, E3cartridge, and E3plate) that can satisfy different sample volumes, concentration, quantities and the need for automation, E3technology could potentially become a truly low-tech and streamlined universal approach for proteomics sample preparation. We further provide an enhanced technology that is capable of processing intact cells in a single device. This single-vessel approach (E4technology) bypasses cell lysis step, further simplifies the conventional proteomic workflow and minimizes sample loss. We understand that the reported numbers of protein and peptide identifications are yet comparable to the ones obtained from established single-cell proteomics methods such as NanoPOT,^34^ SCoPE,^35^ and T-SCP.^36^ However, we believe the data are encouraging, as in contrast to the reported studies that require either extensive sample processing on microfluidics or chips, sophisticated improvements of LC column/method, in-house customization of MS instrumentation,^37^ bioinformatic manipulations of the identifications,^38^ all of which have high technical barrier to general proteomics labs, we employed only a regular single-vessel filter-based sample processing and a typical Orbitrap MS. Our approach is much accessible and affordable, and could be readily incorporated into existing pipelines. In addition, the usefulness of E4technolgy could be expanded by coupling the on-filter in-cell (OFIC) digestion with on-filter TMT labeling,^39^ which certainly deserves further exploration. In summary, we believe E4technology could be a very attractive alternative to proteomic analysis of low cells or single cells.

## Data Availability

All MS raw data associated with the study and the detailed tables of MaxQuant output files have been deposited to the MassIVE server (https://massive.ucsd.edu/) with the dataset identifier MSV000092423 and doi:10.25345/C55T3G928.
